# Tumour hypoxia promotes melanoma growth and metastasis via High Mobility Group Box-1 and M2-like macrophages

**DOI:** 10.1038/srep29914

**Published:** 2016-07-18

**Authors:** Roman Huber, Barbara Meier, Atsushi Otsuka, Gabriele Fenini, Takashi Satoh, Samuel Gehrke, Daniel Widmer, Mitchell P. Levesque, Joanna Mangana, Katrin Kerl, Christoffer Gebhardt, Hiroko Fujii, Chisa Nakashima, Yumi Nonomura, Kenji Kabashima, Reinhard Dummer, Emmanuel Contassot, Lars E. French

**Affiliations:** 1 Department of Dermatology, University Hospital Zürich, Zürich 8091, Switzerland; 2 Skin cancer Unit, German Cancer Research Centre (DKFZ), Heidelberg, Germany; 3 Department of Dermatology, Venereology and Allergology, University Medical Centre Mannheim, Ruprecht-Karl University of Heidelberg, Mannheim, Germany; 4 Department of Dermatology, Kyoto University Graduate School of Medicine, Kyoto, Japan

## Abstract

Hypoxia is a hallmark of cancer that is strongly associated with invasion, metastasis, resistance to therapy and poor clinical outcome. Tumour hypoxia affects immune responses and promotes the accumulation of macrophages in the tumour microenvironment. However, the signals linking tumour hypoxia to tumour-associated macrophage recruitment and tumour promotion are incompletely understood. Here we show that the damage-associated molecular pattern High-Mobility Group Box 1 protein (HMGB1) is released by melanoma tumour cells as a consequence of hypoxia and promotes M2-like tumour-associated macrophage accumulation and an IL-10 rich milieu within the tumour. Furthermore, we demonstrate that HMGB1 drives IL-10 production in M2-like macrophages by selectively signalling through the Receptor for Advanced Glycation End products (RAGE). Finally, we show that HMGB1 has an important role in murine B16 melanoma growth and metastasis, whereas in humans its serum concentration is significantly increased in metastatic melanoma. Collectively, our findings identify a mechanism by which hypoxia affects tumour growth and metastasis in melanoma and depict HMGB1 as a potential therapeutic target.

Hypoxia is a hallmark of a wide range of advanced solid tumours^1^ and is often associated with poor prognosis in cancer patients^2^. Up to 60% of advanced solid tumours exhibit large hypoxic areas as a result of an imbalance between their oxygen supply and consumption^3^. A hypoxic environment, as frequently observed in solid tumours, results in focal areas of tumour cell necrosis and the release of damage associated molecular patterns (DAMPs) and alarmins, a group of endogenous molecules including hyaluronan fragments, S100 molecules and heat shock proteins, amyloid-β, uric acid, IL-1, IL-33 and high-mobility group box 1 protein (HMGB1)^4^.

The non-histone nuclear protein HMGB1, a highly conserved DNA-binding protein with 98.5% sequence homology across mammals^5^,^6^, can be actively secreted by immune cells including macrophages or passively released upon cell damage. HMGB1 has been shown to engage several different receptors including members of the Toll-like receptor (TLR) family^7^ and the Receptor for Advanced Glycation End products (RAGE), thereby inducing an inflammatory response^8^,^9^. HMGB1 has been reported to be overexpressed in various solid tumours, shown to be released by mesothelial cells exposed to asbestos and erionite and implicated in malignant mesothelioma development in an autocrine manner^10^,^11^,^12^. HMGB1 has also been implicated in the development of hepatocellular carcinoma^13^ and both colon and inflammation-related skin cancers^14^,^15^.

Tumour hypoxia has a major impact on the tumour microenvironment and the subsequent immune response. Areas of hypoxia in tumours have been reported to be associated with advanced stage malignancy and resistance to therapy^16^. Paradoxically, tumour hypoxia promotes the recruitment of leukocytes, which can promote tumour cell proliferation, angiogenesis and metastasis^17^,^18^. Among the diverse immune cell populations present in the tumour microenvironment, macrophages are the most abundant^19^ and are referred as Tumour-Associated Macrophages (TAMs). Macrophages differentiate or polarize in response to inflammatory stimuli and secrete a distinctive set of cytokines and amongst the different types of polarized macrophages described to date, the so-called M1-like or M2-like macrophages appear to have opposing effects on tumour progression^20^. M1-like macrophages are associated with type 1 T-cell responses and anti-tumour immune responses, while M2-like macrophages are reported to display tumour-promoting properties including the production of proteolytic enzymes, the suppression of anti-tumour immune responses and the promotion of angiogenesis^21^. Finally, in advanced tumours, TAMs have been reported to be preferentially skewed towards an M2-like phenotype^22^,^23^,^24^.

Given the strong association between tumour hypoxia, tumour progression and poor clinical outcome, deciphering the precise molecular mechanisms by which hypoxia regulates tumour behaviour is of great interest and relevance. Here, we show that hypoxic melanoma cells release the alarmin HMGB1, which promotes tumour growth and metastasis through the accumulation, within tumours, of TAMS bearing an M2-like phenotype.

## Results

### Serum HMGB1 levels are elevated in metastatic melanoma patients

**Figure 1:**
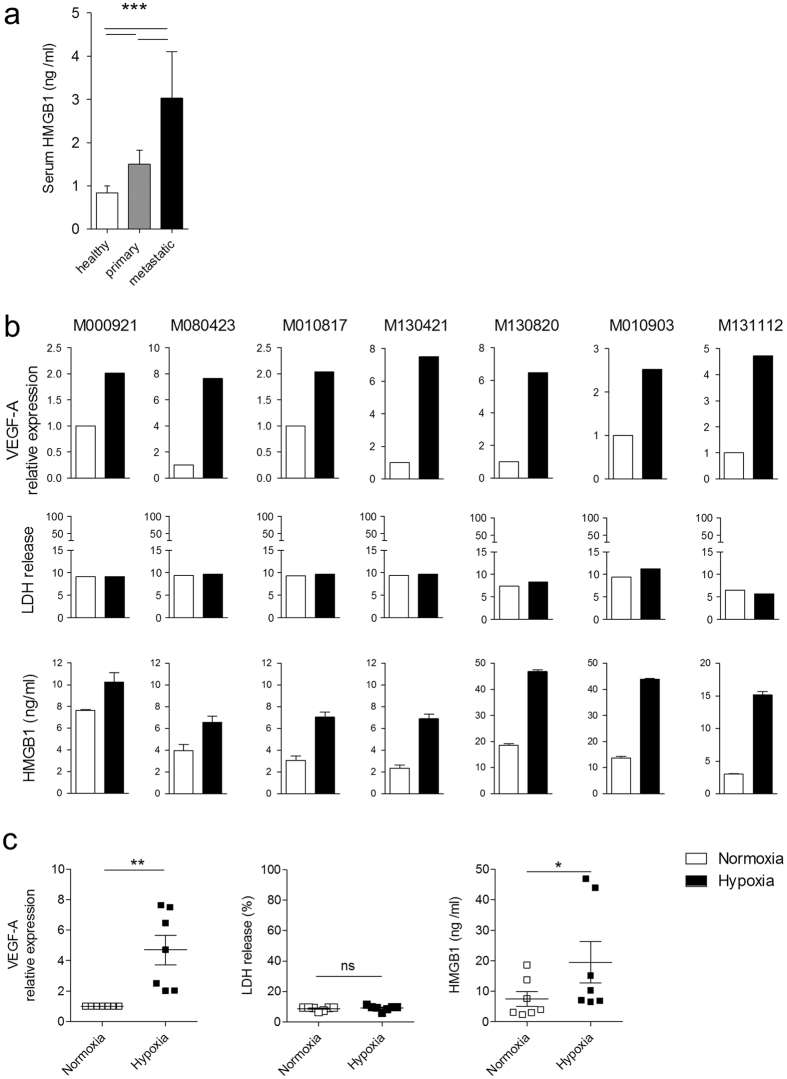
HMGB1 is increased in the serum of patients with metastatic melanoma and released by melanoma cells under hypoxic conditions. (**a**) HMGB1 was measured by ELISA in the serum of healthy individuals (n = 10), and patients with primary (n = 9) or metastatic melanoma (n = 11). Data are expressed as the mean +/− SEM are presented. ***P < 0.001 using ANOVA followed by Turkey test. (**b**) Cell-lines derived from human melanoma metastases were cultured under normoxic or hypoxic conditions for 72 h. As a marker of hypoxia, VEGF-A expression was assessed by qPCR (top panels). Cell viability was assessed in cell culture supernatants with an LDH release assay (middle panels) and HMGB1 release measured by ELISA (bottom panels). (**c**) Statistical representation and analysis of the data presented in (**b**). Data expressed as the mean +/− SEM are presented. A paired Student t-test was performed. *P < 0.05, **P < 0.01.

We first determined whether levels of HMBG1 are altered in cancer patients. To this end, we analysed HMGB1 levels in the serum of patients with primary melanoma, metastatic melanoma and in age-matched healthy volunteers. Significantly, increased levels of HMGB1 were found in the serum of patients with metastatic melanoma when compared to patients with primary melanoma and to healthy donors (Fig. 1a). This observation suggests that the extent of HMGB1 release correlates with the stage of the disease.

### Tumour cell hypoxia drives HMGB1 release *in vitro* and *in vivo*

Hypoxia in the context of liver ischemia and hepatocellular carcinoma has been previously reported to induce HMGB1 release^13^,^25^. To determine if metastatic melanoma cells release HMGB1 in response to hypoxia, we analysed metastatic melanoma cell-lines grown in hypoxic versus normoxic conditions *in vitro*. When compared to their counterparts grown in normoxic conditions, all tested melanoma cell-lines (n = 7) released enhanced levels of HMGB1 when grown in hypoxic conditions in which increased expression of the hypoxia target gene VEGF-A was also observed^26^ (Fig. 1b,c). Notably, under the hypoxic conditions tested no increase in cell mortality was observed as revealed by the consistently low levels of LDH release. The induction of HMGB1 expression by hypoxia in melanoma cells in culture was only significantly induced after 72 hours of hypoxia, but not at shorter exposure times to hypoxia.

**Figure 2:**
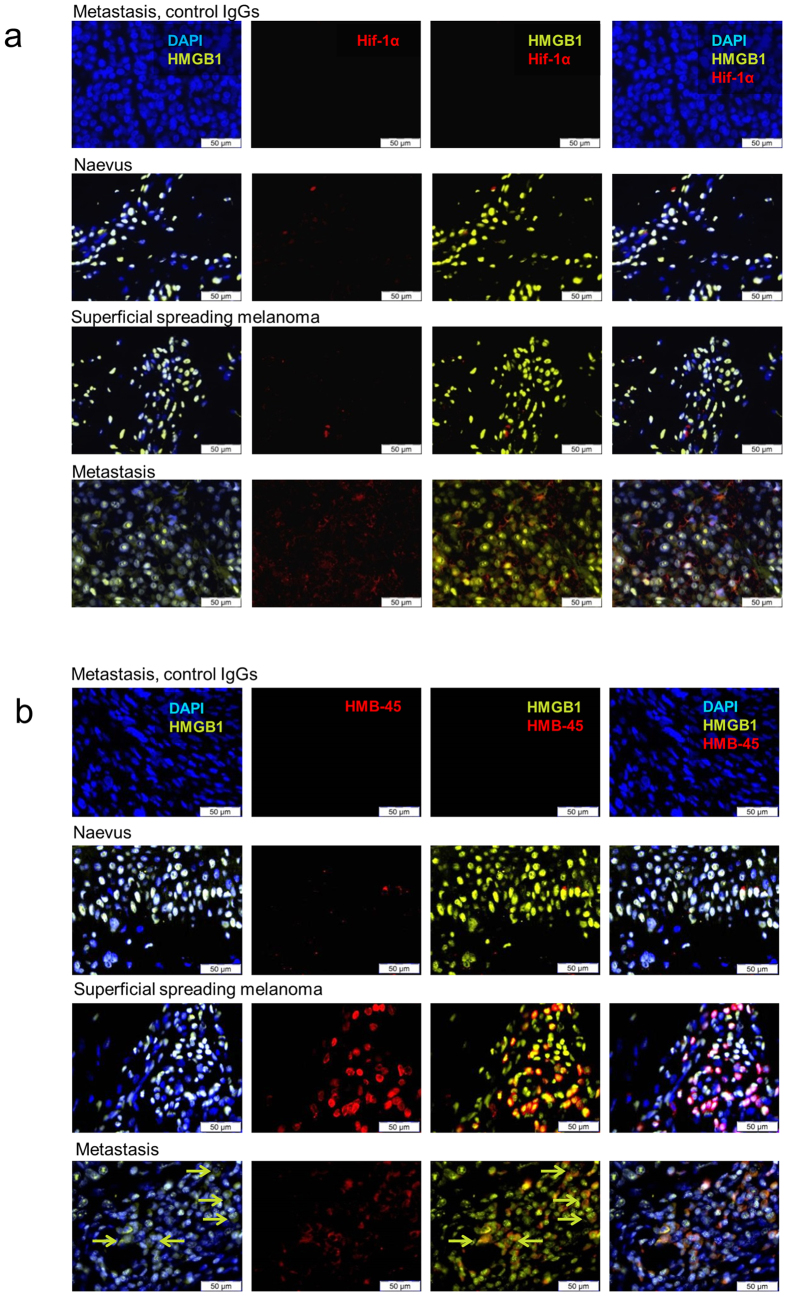
HMGB1 is released by tumour cells in hypoxic tumour areas of human metastatic melanoma. (**a**) Immunofluorescence labelling, using antibodies to HIF1α (red) and HMGB1 (green), was performed on healthy skin, nevi, primary cutaneous melanoma and metastases as indicated (n = 5/group; representative pictures are presented). (**b**) Immunofluorescence labelling using antibodies to human melanosome (clone HMB-45, red) and HMGB1 (green) was performed on healthy skin, nevi, primary cutaneous melanoma and metastases as indicated (n = 5/group; representative pictures are presented).

Under physiologic conditions, HMGB1 is known to be sequestered in the nucleus, whereas cytoplasmic relocalization of HMGB1 has been reported to occur prior to active secretion or passive HMGB1 release^11^. First, we observed that melanoma metastasis cell lines expressed both cytosolic and nuclear HIF1α when cultured under low oxygen conditions, whereas HIF1α was not detectable when cells were kept in normoxic conditions. The co-labelling with an anti-HMGB1 antibody revealed that HMGB1 is located in the cytosol of HIF1α-positive cells in hypoxic conditions whereas HMGB1 was confined to the nucleus of cells kept in normoxic conditions (Fig. S1). When the intracellular localization of HMGB1 was assessed in tissue sections of nevi, primary melanoma (superficial spreading melanoma) and melanoma metastases, nuclear HMGB1 staining was observed in nevi and primary melanomas, whereas in melanoma metastases large areas of the tumour were composed of cells with cytoplasmic HMGB1 staining (Fig. 2a). Interestingly, the latter areas were also Hif1α-positive indicating that HMGB1 is released within hypoxic areas of metastatic melanomas (Fig. 2a). Co-labelling with an anti-melanosome antibody (clone HMB-45) showed that, in metastasis, HMGB1 is released by HMB-45^+^ melanoma cells in contrast to primary tumours where HMGB1 was exclusively found in the nucleus of HMB-45^+^ melanoma cells (Fig. 2b). These observations indicate that HMGB1 is released by hypoxic tumour cells within metastases in melanoma patients.

### HMGB1 release is partly dependent on HIF1α in cells in hypoxic condition

**Figure 3:**
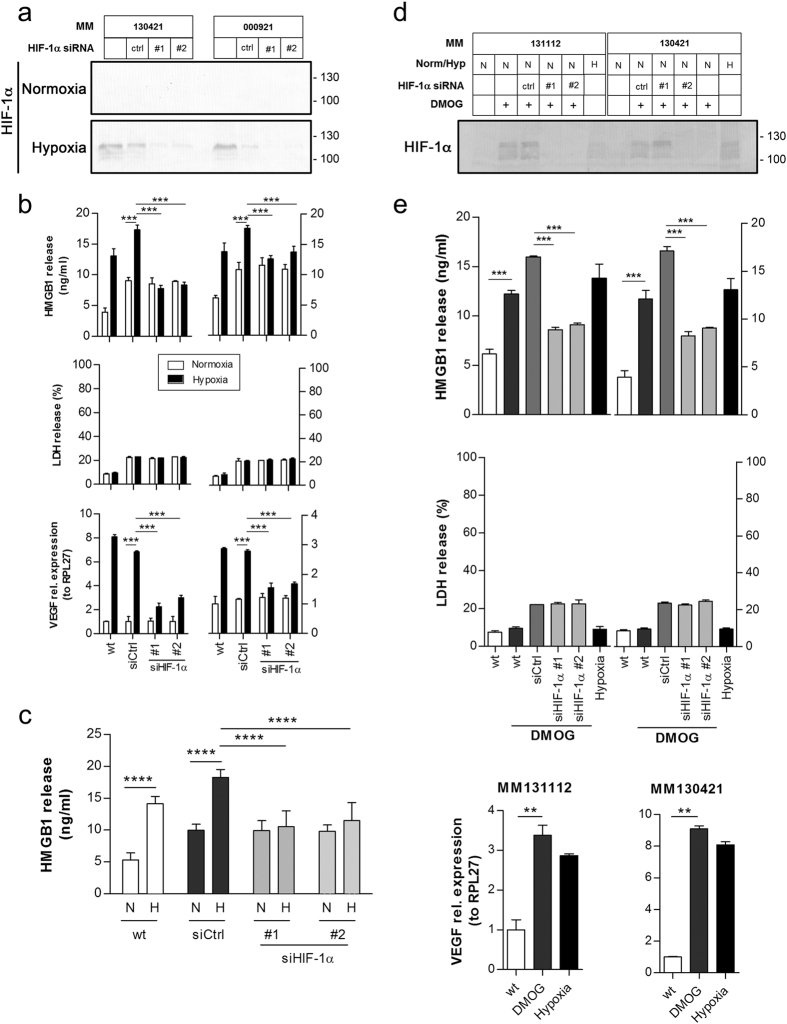
HMGB1 release is dependent on HIF1α. (**a**) HIF1α expression was assess by Western blotting in two human metastatic melanoma cell lines (MM130421 and MM000921) transfected with 2 sequences of siRNA to HIF1α and incubated for 72 hrs in normoxic or hypoxic conditions. (**b**) MM130421 and MM000921 were cultured under normoxic or hypoxic conditions for 72 hrs. As an hypoxia target gene, VEGF-A expression was assessed by qPCR (bottom panels). Cell viability was assessed in cell culture supernatants with an LDH release assay (middle panels) and HMGB1 release measured by ELISA (top panel). (**c**) HMGB1 release summary and statistical analysis of the data presented for individual cell lines in (**b**). Data expressed as the mean +/− SEM are presented. A paired Student t-test was performed. ****P < 0.0001. (**d**) HIF1α expression was assessed by Western blotting in two human metastatic melanoma cell lines (MM130421 and MM131112) transfected with 2 sequences of siRNA to HIF1α and exposed to DMOG or incubated for 72 hrs in normoxic (Nor, N) or hypoxic conditions (Hyp, H). (**e**) HMGB1 release was measured by ELISA (top panels). Cell viability was assessed in cell culture supernatants with an LDH release assay (middle panels) and VEGF-A expression was assessed by qPCR (bottom panels).

To assess whether HMGB1 release under hypoxic culture condition was dependent on HIF1α or not, we knocked HIF1α down using siRNA in two human melanoma cell lines. After seventy-two hours under low oxygen conditions, increased levels of stabilized HIF1α were detected in both cell lines, either unmodified or transfected with a control siRNA (and Fig. 3a). In contrast, little or no expression of HIF1α was detected in both cell lines transfected with 2 independent HIF1α sequences (Fig. 3a). Interestingly, a reduced HMGB1 secretion under hypoxia was observed in both cell lines transfected with both HIF1α-siRNA as measure by ELISA (Fig. 3b,c). Under the hypoxic conditions tested, no increase in cell mortality was observed as revealed by the consistently low levels of LDH release and VEGFA was always found to be upregulated (Fig. 3b). Notably, HMGB1 secretion decrease was of only 50%, suggesting that mechanisms, other than HIF1α, are involved (Fig. 3b,c). By inhibiting oxygen-sensitive prolyl hydroxylases (PHDs) with DMOG, we observed that the resulting increased stabilization of HIF1α (Fig. 3d) was associated with a higher HMGB1 release in melanoma cell lines (Fig. 3e). DMOG exposure of siRNA-transfected melanoma cells did not result in significant increase of HMGB1 release (Fig. 3e), therefore reinforcing the role of the hypoxia/HIF1α axis in HMGB1 secretion.

### HMGB1 promotes melanoma growth and metastasis

**Figure 4:**
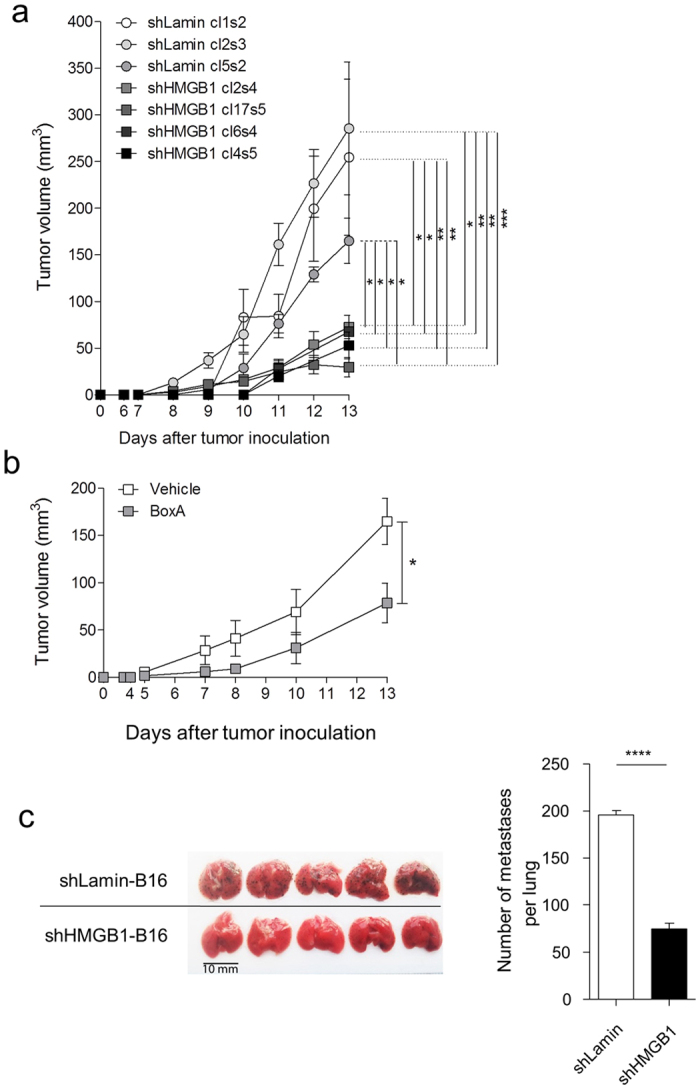
HMGB1 promotes tumour growth and metastasis, and is released by hypoxic tumour cells *in vivo*. (**a**) Tumour growth in C57BL/6 mice subcutaneously injected with 1 × 10^5^ B16 clones transduced with shRNAs specific to HMGB1 or lamin and (**b**) tumour growth in C57BL/6 mice subcutaneously injected with 1 × 10^5^ B16 cells transduced with lamin-shRNA (cl1s2: clone 1, shRNA sequence 2) and treated i.p. every 3 days from day 0 with 50 μg a recombinant HMGB1 inhibitor (BoxA). (**c**) 1 × 10^5^ B16 cells transduced with HMGB1- or lamin-shRNA were injected i.v. to C57BL/6 mice and lung metastases were counted after 13 days. A macroscopic view of 5 representative lungs from each group and the numeration of metastases in the lungs (n = 9) are shown. Results are expressed as the mean +/− SEM. *P < 0.05, **P < 0.01, ***P < 0.001, ****P < 0.0001.

To assess whether HMGB1 plays a functional role in melanoma progression, we diminished HMGB1 expression in B16 melanoma cells using shRNA technology. Four HMGB1-specific shRNA sequences and 2 lamin-specific shRNA sequences were transduced into B16 cells. B16 cells showing the highest downregulation of HMGB1 expression (shRNA sequence 5, Fig. S2a, top panel) were subsequently cloned by limiting dilution and expanded under selection pressure (puromycin). B16 cells transduced with shRNA to lamin showing no variation in HMGB1 expression when compared to wild-type B16 cells (shRNA sequence 2 to lamin, Fig. S2a, top panel) were chosen as controls and were also cloned by limiting dilution and expanded under selection pressure. The silencing of HMGB1 was highly stable during *in vitro* expansion (Fig. S2b) and after 13 days of *in vivo* growth (Fig. S2c). The B16 clones transduced with HMGB1-specific shRNA exhibiting the highest HMGB1 knockdown (Fig. S2a, bottom panel) and the clones transduced with lamin-shRNA showing similar HMGB1 expression when compared to wild-type B16 were then injected s.c. in C57BL/6 mice. Following sub-cutaneous injection, mice implanted with HMGB1-shRNA-transduced B16 clones exhibited significantly reduced tumour growth when compared to mice implanted with the same number of lamin-shRNA-transduced B16 clones (Fig. 4a). Importantly, the transduction of lamin-shRNA did not alter the *in vivo* growth kinetics of B16 tumours when compared to wild-type B16 cells (Fig. S3). Since such a reduced growth was reproducibly obtained irrespective of the shRNA sequence and clonal selection of B16 cells, all experiments described below were performed with one B16 clone transduced with HMGB1-specific shRNA and one B16 clone transduced with lamin-specific shRNA (HMGB1 clone 17 (sequence 5) and lamin clone 1 (sequence 2), respectively). This growth delay was neither due to an intrinsic effect of shRNA transduction or HMGB1 knockdown on growth or apoptosis as revealed by the identical *in vitro* growth and apoptosis of HMGB1- and lamin-shRNA-transduced B16 cells (Fig. S4). To further validate the above observation and assess the contribution of released extracellular HMGB1 on tumour growth, we next treated mice implanted subcutaneously with lamin-shRNA-transduced B16 cells with either a recombinant HMGB1 inhibitor (BoxA, 50 μg i.p. every 3 days) or vehicle (PBS) in an independent set of *in vivo* experiments. In accordance with results observed with HMGB1 silencing using shRNA, systemic treatment of tumour-bearing mice with the HMGB1 inhibitor BoxA also resulted in significantly delayed tumour growth compared to mice exposed to vehicle alone (Fig. 4b).

We next evaluated the role of HMGB1 in metastasis by injecting either HMGB1-shRNA-transduced B16 cells or lamin-shRNA-transduced B16 cells i.v. into wild type mice. After 13 days, mice injected with HMGB1-shRNA-transduced B16 cells also exhibited significantly less lung metastases when compared to mice injected with lamin-shRNA-transduced control cells (Fig. 4c).

**Figure 5:**
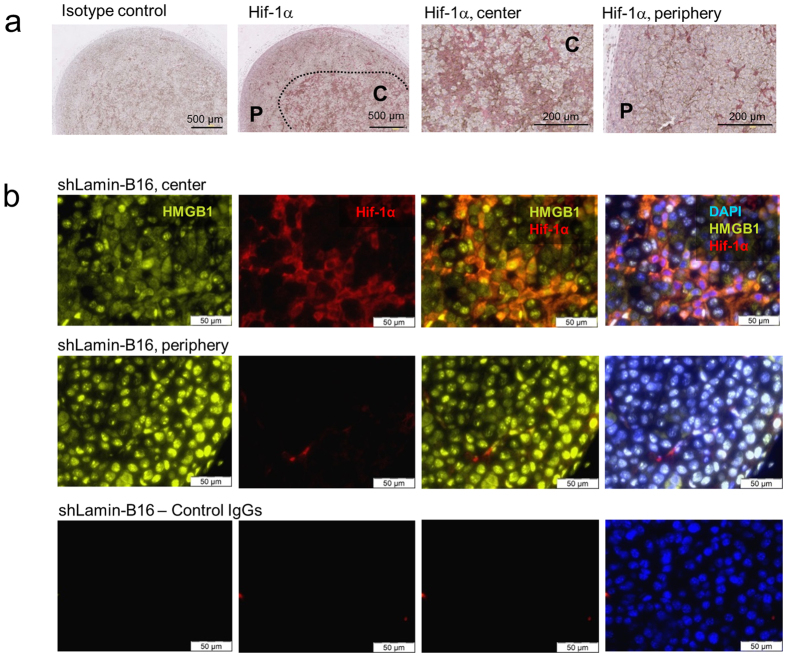
HMGB1 is released in hypoxic regions of B16 tumours. (**a**) Immunohistochemistry using a HIF1α^−^ specific antibody was performed on 200 mm^3^ tumours and revealed large HIF1α^+^ hypoxic areas in the center (C) and HIF1α^−^ normoxic areas in the periphery (P). Representative pictures of 10 tumours are presented. (**b**) Immunofluorescence analysis of B16 tumours transduced with lamin-shRNA using antibodies to HIF1α (red) and HMGB1 (green) showed nuclear HMGB1 localization in normoxic (HIF1α^−^) areas and cytoplasmic HMGB1 localization in hypoxic (HIF1α^+^) areas. Representative pictures of 10 tumours are presented.

To determine whether HMGB1 release in this mouse model of melanoma was reminiscent of what was observed in human melanoma metastases, we assessed the intra-cellular localization of HMGB1 in tumours of mice implanted s.c. with lamin-shRNA-transduced B16 cells. Large areas where HIF was stabilized were found mainly in the centre of B16 tumours, as revealed by immunocytochemistry using a HIF1α antibody (Fig. 5a). Within HIF1α-positive tumour areas, large numbers of tumour cells displaying cytoplasmic HMGB1 expression were observed and double-labelling experiments identified numerous cells co-expressing HMGB1 and HIF1α (Fig. 5b). In contrast, nuclear HMGB1 localisation was predominantly observed in HIF-1α-negative areas of the tumour, and no evident co-labelling of anti-HMGB1 and HIF1α antibodies was observed in such areas. These observations are in line with data from human melanomas. Altogether, these results suggest that HMGB1 released by hypoxic tumour cells promotes melanoma growth and metastasis.

### HMGB1 promotes the accumulation of tumour-associated M2-like macrophages

**Figure 6:**
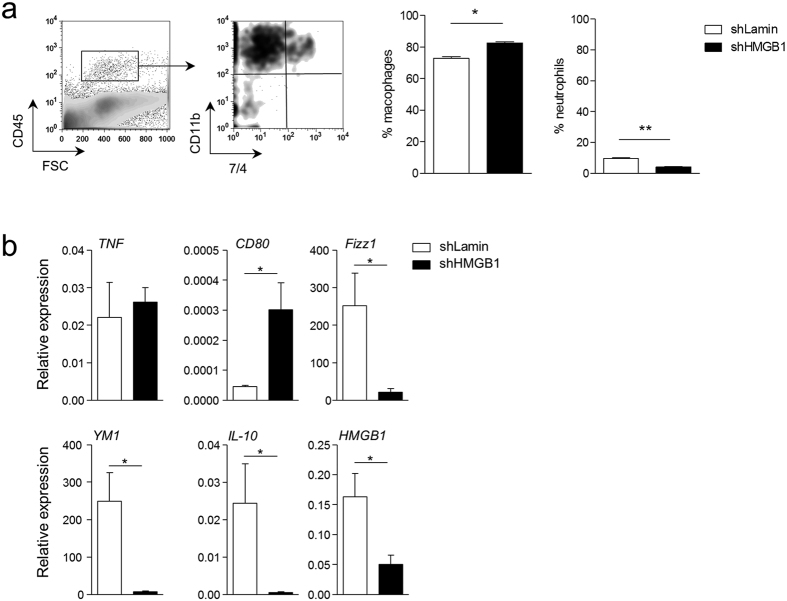
Tumour-derived HMGB1 induces the accumulation of macrophages harbouring an M2-like phenotype. (**a**) Macrophage and neutrophil infiltrates were quantified in 200 mm^3^ tumours. Representative flow cytometry pictures (left panels) and quantification (right panels) of total macrophages and neutrophils in tumours of mice implanted with B16 cells transduced with lamin- (n = 6) or HMGB1-shRNA (n = 6). (**b**) Quantification of M1- and M2-specific gene expression by qPCR in size-matched (200 mm^3^) primary tumours from mice implanted with B16 cells transduced with HMGB1- (shHMGB1, n = 5) or lamin-shRNA (shLamin, n = 5). Results are presented as the mean +/− SEM. *P < 0.05, **P < 0.01.

To further investigate the tumour-promoting effects of HMGB1 release, as a consequence of tumour hypoxia and given the absence of evidence for a direct effect of HMGB1 on tumour cell growth or apoptosis *in vitro*, we analysed the immune cell infiltrate within the tumour microenvironment. Analysis of dissociated tumours by flow-cytometry revealed a significant increase in the total number of TAMs and a slight but significant decrease in the total number of neutrophils in HMGB1-shRNA-transduced B16 tumours (Fig. 6a). To assess the phenotype of these TAMs, we analysed the *in vivo* expression of markers discriminating M1-like and M2-like macrophage subpopulations^27^. Quantitative PCR of macrophages accumulating at the tumour site revealed that the downregulation of HMGB1 in B16 cells was associated with a significant induction of the expression of the M1 macrophage marker *CD80*^28^, whereas lamin-shRNA-transduced B16 tumours were associated with a significant upregulation of the M2 markers *YM1, Fizz1, and IL-10* (Fig. 6b). These results suggest that HMGB1 expression and release within tumours favours the accumulation of M2-like macrophages in the microenvironment.

### HMGB1 induces IL-10 in M2-like macrophages through RAGE

**Figure 7:**
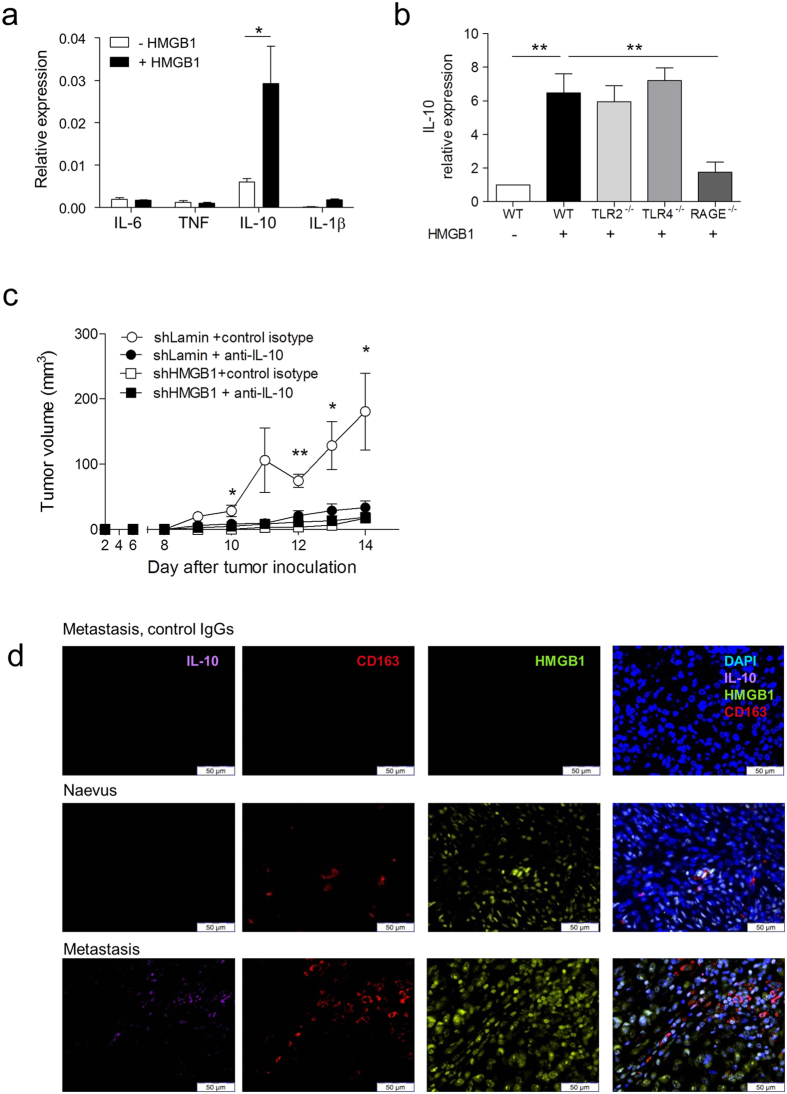
HMGB1 and RAGE-dependent IL-10 production by TAMs favour melanoma growth. (**a**) *IL-6*, *TNF*, *IL-10* and *IL-1β* mRNA expression, relative to housekeeper *RPL27*, in *in vitro*-generated murine M2-like macrophages in the presence or absence of recombinant HMGB1. (**b**) *IL-10* mRNA expression in *in vitro*-differentiated M2-like macrophages from wild-type (WT)*, Tlr2*^−/−^*, Tlr4*^−/−^ and *Rage*^−/−^ mice stimulated with recombinant HMGB1. Expression is relative to untreated WT M2-like macrophages. (**c**) Tumour growth in C57BL/6 mice having received 1 × 10^5^ B16 cells transduced with either HMGB1- or lamin-shRNA and treated with an anti-IL-10 blocking antibody or a control isotype (n = 7 mice/group). (**d**) Immunofluorescence analysis of human biopsies of nevi and melanoma metastases using anti-CD163, anti-HMGB1 and anti-IL-10 antibodies (n = 5/group) revealing IL-10 secretion by TAMs in regions with cytoplasmic (secreted) HMGB1. Representative pictures are shown.

Given the reported role of the cytokine milieu of the tumour microenvironment on tumour progression^29^,^30^, we evaluated the effect of HMGB1 on TAM cytokine expression. While HMGB1 had no effect on IL-6, TNF or IL-1β expression, it significantly increased IL-10 expression in bone marrow-derived M2-like macrophages (BMM2, Fig. 7a). Furthermore, HMGB1-induced upregulation of IL-10 expression was not observed in BMM2 from RAGE^−/−^ mice while it was retained in TLR2- and TLR4-deficient BMM2 (Fig. 7b), suggesting that HMGB1 induces IL-10 in TAMs through RAGE-dependent signalling. To assess the effect of IL-10 on tumour development in the model used herein, mice implanted with lamin-shRNA transduced B16 tumours were treated with an anti-IL-10 neutralizing antibody. Similar to mice in which tumour expression of HMGB1 was silenced or inhibited with recombinant BoxA, mice treated with anti-IL-10 exhibited significantly delayed tumour growth compared to controls, whereas anti-IL-10 did not significantly affect the growth of HMGB1-shRNA-transduced B16 tumours (Fig. 7c). Collectively, these results indicate that HMGB1-dependent production of IL-10 by tumour-associated M2-like macrophages contributes to tumour progression in our mouse melanoma model. In further support for a potential role of TAMs and IL-10 in melanoma is the presence of IL-10-producing TAMs in human melanoma metastases as revealed by the presence of CD163^+^ IL-10^+^ cells infiltrating human metastases in areas with high cytoplasmic HMGB1 expression (Fig. 7d). In contrast, lower expression and nuclear localisation of HMGB1 in nevi was associated with a very discrete presence of CD163^+^ cells and an absence of IL-10 production. Taken together, these results are supportive of a tumour-promoting role for HMGB1 dependent on its ability to induce IL-10 secretion by M2-like macrophages in a RAGE-dependent manner.

## Discussion

Tumour-associated macrophages (TAMs) have emerged as key components of the tumour microenvironment with a crucial role in tumour progression^31^. TAMs are reported to be associated with poor prognosis in several types of cancer^32^,^33^,^34^,^35^, and it has been proposed that monocytes, which continuously infiltrate tumours, once polarized to M2-like macrophages preferentially accumulate in hypoxic tumour areas^36^,^37^,^38^,^39^,^40^. By promoting angiogenesis and metastasis, M2-polarized TAMs can “assist” the tumour in overcoming a hostile hypoxic environment and thus sustain its progression^24^,^41^. Therapeutic targeting of this process in cancer is currently limited by an inadequate understanding of factors released by hypoxic cells that may be associated with M2-like macrophage accumulation in the tumour microenvironment. Here, we show that the alarmin HMGB1, which is released from tumour cells under hypoxic conditions, plays a critical role in promoting tumour progression by triggering the accumulation of M2-like TAMs. We demonstrate that HMGB1 released by hypoxic tumour cells favours the accumulation of M2-like macrophages at the tumour site and their secretion of IL-10. The relevance of the *in vitro* and *in vivo* data generated in this mouse model of melanoma is substantiated by the observation that HMGB1 release also occurs in human melanoma cell-lines under hypoxic conditions *in vitro*, particularly in Hif-1α-positive areas within human melanoma metastases and by the fact that melanoma metastases but not benign melanocytic nevi are infiltrated by CD163^+^ IL-10^+^ TAMs.

Areas of hypoxia in tumours have been reported to be associated with advanced stage malignancy and resistance to therapy^16^. Whether hypoxia and its by-products directly induce an M2-like phenotype in TAMs remains unclear. Our data suggest that, although HMGB1 clearly contributes to the accumulation of M2-like macrophages within tumours, it does not appear to be directly involved in M2 polarization. Indeed, and in line with a previous report showing that hypoxia is not a driver of the differentiation of TAMs^37^, we were not able to differentiate macrophages towards an M2-like phenotype *in vitro* using recombinant HMGB1 alone. However, a recent study showed that lactic acid, produced by tumour cells as a by-product of hypoxic glycolysis, has a critical function in tumour development by inducing VEGF expression and M2-like polarization of TAMs in a HIF1α-dependent manner^42^. Furthermore, oncostatin M and eotaxin have been suggested to promote breast cancer metastasis by favouring M2 polarization and tumour infiltration^43^. Recent evidence also suggests that HMGB1 may directly act on progenitor cells to favour the induction of myeloid-derived suppressive cells^44^.

Mechanistically, hypoxia-induced HMGB1 release seems to be associated with several tumour-promoting events. In advanced hepatocarcinomas, hypoxia has also been shown to be associated with the release of HMGB1^45^, and it has been suggested that HMGB1, via RAGE, induces the expression of NF-κB-dependent pro-angiogenic factors including VEGF^46^ and the matrix metalloproteinases MMP2 and MMP9^47^. It has also been observed that HMGB1 released from dying cells in prostate cancer induces the accumulation of tumour-infiltrating T cells and the expression of lymphotoxin-α1β2 on their surface, which in turn recruits macrophages to the tumour and supports angiogenesis^48^. Noteworthy, tumour cells or tumour-infiltrating immune cells seem not to be the sole source of HMGB1. Indeed, UVB radiation of the skin has been shown to induce HMGB1 release from epidermal keratinocytes, resulting in a neutrophilic inflammatory response that stimulate angiogenesis and promotes melanoma metastasis in mice^49^.

We also observed that HMGB1 directly induced the production of IL-10 in TAMs and that blockade of IL-10 with a neutralizing antibody led to delayed tumour growth in the B16 mouse melanoma model. As we cannot, at present, technically and specifically delete IL-10 in TAMs, the relative role of HMGB1-induced IL-10 in TAMs remains incompletely elucidated in our model. It is known that regulatory T cell-mediated/IL-10-dependent suppression of CD8^+^ T cells can be blocked by removal of tumour-derived HMGB1^50^, which is consistent with our observation that HMGB1 inhibition leads to delayed tumour growth although via an alternate mechanism. It is likely that IL-10, which can also be produced by melanoma cells^51^ and tumour-associated myeloid-derived suppressor cells^44^ may favour immunoregulatory responses by inducing the downregulation of molecules involved in antigen presentation to CD8^+^ T cells^52^, by inducing regulatory T cells^53^,^54^ and/or by supressing the production of pro-inflammatory cytokines including TNFα, IFN-γ and IL-2 by T cells^55^. In accordance with this, elevated IL-10 production levels in melanoma patients are associated with poor prognosis^56^.

HMGB1 can signal by binding to TLR2, -4 and -9 as well as RAGE. According to our data, TLR signalling was not required for HMGB1-dependent induction of IL-10, the latter being selectively dependent on RAGE signalling. The potential importance of the HMGB1-RAGE interaction in promoting tumour progression is supported by a recent report showing that RAGE and HMGB1 are associated with the progression of prostate cancer and poor patient outcome^57^. However, since our data shows that HMGB1 downregulation or the use of a soluble inhibitor did not lead to complete inhibition of tumour growth, it is very likely that signalling by other alarmins through TLRs or RAGE also have the ability to induce a tumour-promoting microenvironment^58^. In addition, we do not exclude an incomplete inhibition of the effect of HMGB1 in our model or other HMGB1-independent mechanisms of tumourigenesis.

In conclusion, we demonstrate that HMGB1, derived from hypoxic tumour cells, significantly contributes to melanoma progression by favouring the accumulation of IL-10-secreting TAMs within the tumour. Tumour-derived HMGB1 released as a consequence of focal intra-tumoural hypoxia thus directly contributes to tumour progression and likely represents an attractive therapeutic target for tumour therapy as demonstrated here in the case of melanoma.

## Methods

### Biological samples from melanoma patients and healthy donors

Serum and tumour biopsies were collected from patients with primary melanoma or metastatic melanoma (stage IIIB to IV) in the Department of Dermatology of the University Hospital of Zürich. Characteristics of patients with primary and metastatic melanoma are reported in Tables 1 and 2, respectively. Serum was obtained from healthy blood donors and healthy skin was obtained as excess skin resulting from aesthetic/reconstructive procedures in the plastic surgery unit at the University Hospital of Zürich. All human biological samples were collected after written informed consent of the patient and with approval of Local Ethics Committee (Kantonale Ethikkommission Zürich, KEK-ZH authorization Nr. 2014-0425) in accordance to GCP guidelines and the Declaration of Helsinki.

### Mice

Six to 8-week-old female C57BL/6 mice (Harlan, Venray, Netherlands) were used in this study. TLR2^−/−^ C57BL/6 mice were kindly provided by Prof. Marc Donath (Department of Biomedicine, University Hospital Basel, Switzerland), TLR4^−/−^ C57BL/6 mice were kindly provided by Prof. Markus G. Manz (Division of Haematology, University Hospital Zürich, Switzerland) and RAGE^−/−^ C57BL/6 mice were kindly provided by Prof. Peter P. Nawroth (Department of Internal Medicine and Clinical Chemistry, University of Heidelberg, Germany). All experimental procedures were approved by the Veterinary Office of Zürich and the institutional animal care officer and were carried out in accordance to the approved guidelines.

### Generation of HMGB1-shRNA-transduced B16

B16-F10 mouse melanoma cell-lines stably expressing shRNA specific to Lamin or HMGB1 were generated by transducing B16-F10 cells with a lentiviral vector. Briefly, specific shRNA were generated by inserting oligonucleotides targeting Lamin or HMGB1 into the pSUPER vector (Oligoengine, Seattle, WA) and subsequent cloning into the lentiviral vector pSP-93 (Oligoengine). Second-generation packaging plasmids pMD2-VSVG and psPAX2 (kindly provided by Prof. J. Tschopp, Biochemistry Institute, Lausanne, Switzerland) were used for lentivirus production and infection. For each target molecule, the procedure was performed with different shRNA sequences. ShRNA-transduced B16 cells were subsequently cloned by limiting dilution under selection pressure (puromycin). For each shRNA, one clone was chosen according to knock-down efficiency and stability as well as *in vitro* properties (Figs S1 and S3).

### Generation of HIF1α-siRNA-transduced human melanoma cell lines

siRNA transfection of metastatic melanoma cells was carried out using INTERFERin transfection solution according to the manufacturer’s protocol (Polyplus Transfection, Illkirch, France). Cells were transfected with 10 nM of siRNA to Hif1α two hours before hypoxia treatment for 72 hours before supernatant was harvested and RNA or protein was extracted. All-Star negative siRNA sequence (Qiagen) was used as control siRNA. The following siRNAs were used:

siHIF-1α#1: Hs_HIF1A_5 (NM_001530; S102664053, Qiagen FlexiTube).

siHIF-1α#2: Hs_HIF1A_6 (NM_001530; S102664431, Qiagen FlexiTube).

### Tumour growth and metastasis experiments

Lamin-shRNA-transduced or HMGB1-shRNA-transduced B16 cells (1 × 10^5^) were injected subcutaneously in 100 μl PBS into the flank of wild-type mice. Where indicated, mice were treated with 50 μg BoxA (HMGBiotech, Milano, Italy), one of the highly conserved DNA-binding domains of HMGB1, which antagonizes HMGB1 binding to its receptor RAGE. In independent sets of experiments, 50 μg anti-IL-10 antibody or control isotype (Biolegend, San Diego, CA) was injected intraperitoneally every 3 days in 100 μl PBS. Mice were monitored every other day and tumour size was measured using a microcaliper (Mitutoyo, Kawasaki, Japan). Tumour volume is expressed as (long diameter × shorter diameter^2^) × π/6. Lung metastases were generated by tail vein intravenous injection of 1 × 10^5^ B16 cells in 100 μl of PBS. Mice were sacrificed 13 days after tumour cell inoculation and the number of macroscopically visible melanoma metastases on the surface of the lungs was counted in a blinded manner by two different investigators.

### Flow cytometry analysis of tumour-infiltrating cells

Minced tumours were incubated at 37 °C for 60 min in complete RPMI containing 1.5 mg/ml collagenase D (Roche Diagnostics, Rotkreuz, Switzerland). The resulting cell suspensions were filtered through a 40 μm cell strainer (Corning Inc., New York, NY) and stained with anti-mouse 7/4 antibody (Abcam, Cambridge, UK), anti-mouse CD11b and CD45 antibodies (Biolegend). The absolute numbers of each cell subset were calculated by flow cytometry and presented are the numbers per mm^3^ of tumour. Flow-cytometry analysis was performed with a FACS Canto II (Becton-Dickinson, Franklin Lakes, NJ) and FACS DIVA software (Becton-Dickinson).

### Immunohistochemistry and immunofluorescence

To analyze Hif-1α expression in immunohistochemistry, 2 μm paraffin-embedded sections were stained overnight at 4 °C with a monoclonal anti-mouse Hif-1α Ab (Abcam) or an isotype IgG control Ab (Abcam). Samples were incubated for 60 min at room temperature with biotinylated goat anti-mouse IgG (Abcam) coupled to streptavidin-alkaline phosphatase (Vector Laboratories, Burlingame, CA) for 45 min at room temperature. Alkaline phosphatase activity was revealed with the DAKO real detection system (DAKO, Glostrup, Denmark) following the manufacturers’ instructions. After counter-staining with Haematoxylin solution (Sigma, Zug, Switzerland), slides were mounted with Fluorescence Mounting Medium (DAKO) and scanned with a digital slide scanner (Hamamatsu Photonics, Hamamatsu, Japan). Pictures were analysed with the ImageJ software (open source from the National Institutes of Health). For immunofluorescence analyses, 2 μm paraffin-embedded sections were stained overnight at 4 °C with 1 μg/ml rabbit polyclonal anti-mouse HMGB1 Ab (Abcam) and 10 μg/ml mouse monoclonal anti-mouse Hif-1α Ab (Abcam) or alternatively 714 μg/ml monoclonal anti-human melanosome (clone HMB-45, DAKO) or with control IgG antibodies (Abcam) at corresponding concentrations. To determine IL-10 and CD163 expression together with HMGB1sections were stained overnight at 4 °C with rabbit polyclonal anti-human IL-10 (R&D Systems), monoclonal anti-human CD163 (Leica Biosystems, Wetzlar, Germany) and HMGB1 (Abcam) or with respective control IgG antibodies. Samples were then incubated for 60 min at room temperature with conjugated secondary antibodies as follows: Alexa Fluor^®^ 546 Goat Anti-Rabbit IgG (Life Technologies) for HMGB1 and IL-10, and DyLight^®^ 650 Goat Anti-Mouse IgG (Abcam) for Hif-1α and CD163, and Alexa Fluor^®^ 647 Donkey Anti-Goat IgG H&L (Abcam) for IL-10. Slides were mounted with Fluorescence Mounting Medium (DAKO) and analysed with a Widefield BX61 fluorescence microscope (Olympus, Tokyo, Japan) using the Analysis Pro software (Soft Imaging Systems, Münster, Germany).

### Cell culture in hypoxic conditions and treatment with DMOG

To determine the release of HMGB1 under hypoxic conditions *in vitro*, primary cell cultures from metastatic melanoma patients were cultured under hypoxia. One million cells were cultured in 2.5 ml complete RPMI medium. After an overnight incubation in standard conditions allowing the cells to adhere, cells were incubated in a MIC-101 Modular Incubator Chamber (Billups-Rothenberg, Del Mar, CA), flushed with 20 l/min of certified pre-mixed gas composed of 1% O_2_, 5% CO_2_, and 94% N_2_ (Carbagas, Guemligen, Switzerland). The O_2_ concentration inside the chamber was measured with a disposable VTI-122 Polaro-graphic oxygen cell oxygen sensor (Vascular Technology, Nashua, NH). The hypoxia chamber was placed in an incubator at 37 °C for 72 h and samples were harvested on ice for analysis.

Alternatively, to stabilize HIF1α expression in metastatic melanoma cells, cells were treated with 1 mM Dimethyloxalylglycine (DMOG, Sigma-Aldrich, St Louis, MO) for 72 hours.

### Quantitative PCR analysis and quantitative PCR array

For tumour mRNA isolation, tumours excised from mice were cut into small pieces and incubated for 60 min in lysis buffer (Qiagen, Hilden, Germany) with iron beads. RNA was isolated using an RNeasy kit (Qiagen). For mRNA isolation from *in vitro* cell cultures, supernatants were discarded and cells were washed twice with cold PBS. RNA was isolated using an RNeasy kit (Qiagen) according to the manufacturers’ instructions. Total RNA was converted into cDNA with the RevertAid First Strand cDNA Synthesis Kit (Thermo Fisher Scientific, Waltham, MA). Quantitative RT-PCR was performed with a ViiA™ 7 Real-Time PCR System (Life Technologies, Carlsbad, CA) using FastStart Universal SYBR Green Master (Roche). The PCR included an initial denaturation at 95 °C for 10 min, followed by 40 cycles of 95 °C for 10 s and 58 °C for 30 s. After 15 s at 95 °C, 20 s at 60 °C and 15 s at 95 °C, samples were kept at 4 °C. Expression of mRNA (relative) was normalized to the expression of RPL27 mRNA by the change in cycling threshold (ΔC_T_) method and calculated as 2^−ΔΔCT^. The following primers were used:

Human RPL27: forward 5′-ATCGCCAAGAGATCAAAGATAA-3′, reverse 5′-TCTGAAGACATCCTTATTGACG-3′, Human VEGF-α: forward 5′-TACCTCCACCATGCCAAGTG-3′, reverse 5′-GATGATTCTGCCCTCCTCCTT-3′, Mouse CD80: forward 5′-TCAGTTGATGCAGGATACACCA-3′, reverse 5′-AAAGACGAATCAGCAGCACAA-3′, Mouse Fizz1: forward 5′-CCAATCCAGCTAACTATCCCTCC-3′, reverse 5′-ACCCAGTAGCAGTCATCCCA-3′, Mouse HMGB1: forward 5′-GGCGAGCATCCTGGCTTATC-3′, reverse 5′-GGCTGCTTGTCATCTGCTG-3′, Mouse IL-1β: forward 5′-ATCTTTTGGGGTCCGTCAACT-3′, reverse 5′-GACAGCACACATTTGCAGCTC-3′, Mouse IL-6: forward 5′-TAGTCCTTCCTACCCCAATTTCC-3′, reverse 5′-TTGGTCCTTAGCCACTCCTTC-3′, Mouse IL-10: forward 5′-GCTCTTACTGACTGGCATGAG-3′, reverse 5′-CGCAGCTCTAGGAGCATGTG-3′, Mouse RPL27: forward 5′-AAAGCCGTCATCGTGAAGAAC-3′, reverse 5′-GCTGTCACTTTCCGGGGATAG-3′, Mouse TNF-α: forward 5′-CCCTCACACTCAGATCATCTTCT-3′, reverse 5′-GCTACGACGTGGGCTACAG-3′, Mouse Ym1: forward 5′-AGAAGGGAGTTTCAAACCTGGT-3′, reverse 5′-GTCTTGCTCATGTGTGTAAGTGA-3′.

### Detection of HMGB1 by ELISA

To determine the concentration of HMGB1 in the serum of healthy donors, primary melanoma patients and melanoma patients with metastasis, serum samples were analysed using the HMGB1 ELISA kit (IBL International, Hamburg, Germany) according to the manufacturers’ instructions. To determine the concentration of released HMGB1 from *in vitro* cell cultures, supernatants were centrifuged at 1,500 g for 5 min at 4 °C and analysed using the HMGB1 ELISA kit (IBL International) according to the manufacturers’ instructions.

### *In vitro* differentiation of M1- and M2-like macrophages from bone marrow cells

Recombinant cytokines were all from Peprotech (Rocky Hill, NJ). To generate of M1 and M2 macrophages, bone marrow cells from the tibia and fibula of C57BL/6 mice (Harlan) were cultured at 37 °C in 5% CO_2_ in RPMI supplemented with 1% L-glutamine and 10% foetal bovine serum with 10 ng/ml mouse M-CSF. Medium was replaced on days 3 and 6 and cells were harvested on day 8. For M1 phenotype induction, cells were stimulated for 24 h with 10 ng/ml M-CSF and 100 ng/ml IFN-γ and for an additional 24 h with 10 ng/ml M-CSF and 20 ng/ml ultra-pure LPS (Invitrogen, Carlsbad, CA). For M2 phenotype induction, cells were stimulated twice for 24 h with 10 ng/ml M-CSF and 20 ng/ml IL-4. To determine the effect of HMGB1 on M1 and M2 macrophages, culture medium used to induce M1- or M2-like macrophages was supplemented with 1 μg/ml recombinant HMGB1 (HMGBiotech).

### Statistical analyses

Unless otherwise indicated, data are presented as the means ± standard error of the mean (SEM) and are representative of three independent experiments. Statistical analyses were performed using the Prism Software (GraphPad Software, San Diego, CA). *P*-values were calculated with paired or unpaired Student’s *t* test. Where indicated, statistical analyses where performed using ANOVA test followed by a Tukey test. Differences were considered significant when: *P ≤ 0.05, **P ≤ 0.01, ***P ≤ 0.001 and ****P ≤ 0.0001.

## Additional Information

**How to cite this article**: Huber, R. *et al*. Tumour hypoxia promotes melanoma growth and metastasis via High Mobility Group Box-1 and M2-like macrophages. *Sci. Rep.* **6**, 29914; doi: 10.1038/srep29914 (2016).

## Supplementary Material

Supplementary Information

## Figures and Tables

**Table 1:** Characteristics of patients with primary cutaneous melanoma.

Patient number	Date of Birth	Gender	Melanoma type	Localization	Breslow (mm)
1	24.04.1941	M	NMM	Right upper arm	1.6
2	19.05.1945	M	NMM	Left lower arm	3
3	13.01.1980	M	SSM	Left upper arm	0.9
3	10.08.1944	M	SSM	Left-side chest	1.42
4	12.05.1957	F	SSM	Left upper arm	1.77
5	23.09.1956	F	SSM	Right shoulder blade	1.06
6	14.10.1935	M	SSM	Right lower leg	4
7	14.09.1968	F	SSM	Right shoulder blade	0.82
8	04.03.1980	M	SSM	Abdomen	0.68
9	02.05.1946	F	SSM	Back	1.1

All the patients were Caucasian. (SSM: superficial spreading melanoma; NMM: nodular melanoma).

**Table 2:** Characteristics of patients with metastatic melanoma.

Patient number	Date of birth	Gender	Stage
1	01.05.1934	M	IV
2	02.05.1964	M	IV
3	24.11.1961	M	IV
4	17.07.1958	M	IIIB
5	09.10.1960	M	IV
6	17.04.1971	M	IIIC
7	25.10.1938	M	IV
8	30.06.1947	F	IV
9	21.11.1979	M	IV
10	09.05.1943	M	IV
