# Supplementary material for: Tumour hypoxia promotes melanoma growth and metastasis via High Mobility Group Box-1 and M2-like macrophages
Source: Sci Rep. 2016 Jul 18;6:29914. doi: 10.1038/srep29914 (PMC4947927; doi:10.1038/srep29914)
Supplement: Supplementary Information [file srep29914-s1.doc]

**Supplementary material**

Tumour hypoxia promotes melanoma growth and metastasis via High Mobility Group Box-1 and M2-like macrophages

Roman Huber^1,†^, Barbara Meier^1^, Atsushi Otsuka^1,†^, Gabriele Fenini^1^, Takashi Satoh^1^, Samuel Gehrke^1^, Daniel Widmer^1^, Mitchell P Levesque^1^, Joanna Mangana^1^, Katrin Kerl^1^, Christoffer Gebhardt^3,4^, Hiroko Fujii^2^, Chisa Nakashima^2^, Kenji Kabashima^2^, Yumi Nonomura^2^, Reinhard Dummer^1^, Emmanuel Contassot^1,‡,*^, and Lars E. French^1,‡,*^

1. Department of Dermatology, University Hospital Zürich, Zürich 8091, Switzerland
2. Department of Dermatology, Kyoto University Graduate School of Medicine, Kyoto, Japan
3. Skin cancer Unit, German Cancer Research Center (DKFZ), Heidelberg, Germany
4. Department of Dermatology, Venereology and Allergology, University Medical Center Mannheim, Ruprecht-Karl University of Heidelberg, Mannheim, Germany

**Content:**

- **Supplementary methods**
  - Control of knock-down efficiency and stability
  - *In vitro* cell proliferation and apoptosis
- **Supplementary figures**
  - **Fig S1. Assessment and validation of HIF1α and HMGB1 detection and localization by immunofluorescence.**
  - **Fig. S2.** Selection of HMGB1 knock-downs and stability of silencing efficiency over time *in vitro* and *in vivo*
  - **Fig. S3.** Validation of B16 cells transduced with lamin-specific shRNA as control.
  - **Fig. S4.** The *in vitro* growth properties of B16 cells transduced with shRNA specific to lamin or HMGB1 are identical.

**Supplementary methods**

**Control of knock-down efficiency and stability**

To determine the knock-down stability of the B16-F10 mouse melanoma cell-line transduced with shRNA specific to HMGB1, shHMGB1-B16 as well as shLamin-B16 were cultured at 37°C in 5 % CO_2_ in cDMEM medium (DMEM supplemented with 1 % L-glutamine and 10 % fetal bovine serum) and 1x10^5^ cells were lysed at day 0, 7, 14, 21 and 28 in 10mM Tris pH 7.5, 1% NP-40, 150mM NaCl, 5mM EDTA with protease inhibitors (Roche). The cells lysates were subjected to Western-blotting using a rabbit polyclonal anti-β-actin (Cell Signaling) or a rabbit polyclonal anti-HMGB1 antibody (Abcam). Secondary antibodies were coupled to alkaline phosphatise (AP). AP detection was performed using the NBT/ BCIP substrate kit (Promega, Madison, WC). Blots were scanned using the CanonScan 9950F scanner (Canon, Tokyo, Japan).

***In vitro* cell proliferation and apoptosis**

To compare *in vitro* proliferation of B16-F10 stably expressing shRNA specific to HMGB1 or lamin, 1.5 ×10^5^ cells were cultured at 37°C in 5 % CO_2_ in DMEM supplemented with 1 % L-glutamine and 10 % fetal bovine serum (cDMEM). At days 0, 1, 2 and 3 mitochondrial dehydrogenase activity of living cells was measured by incubation with 10 % MTT (Sigma-Aldrich) for 4 h at 37°C. Optical densities were measured by the SpectraMax190 plate reader (Molecular Devices).

Proliferation was measured using CFSE. Cells were incubated with PBS + 1 µM CFSE (Thermo Fisher Scientific, Waltham, MA) for 10 min at 37°C and seeded in 6-well plates. At days 0, 3, 6 and 8 cells were detached and CFSE-related fluorescence intensity was determined by flow cytometry. Acquisition was performed with a FACS Canto II (Becton-Dickinson) and sample analysis was done using the FACS DIVA software (Becton-Dickinson).

To assess apoptosis in transfected cells by flow cytometry, cells were detached at day 0, 3, 6 and 9 and stained with 1.0 µg/ml propidium iodide (Sigma-Aldrich) and Annexin V (Becton-Dickinson) for 15 min on ice. Acquisition was performed with a FACS Canto II (Becton-Dickinson) and analysed using the FACS DIVA software (Becton-Dickinson).

S**upplementary figures**

**
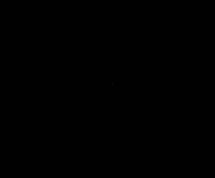

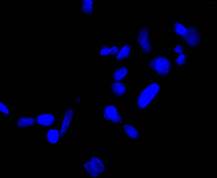

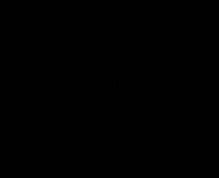

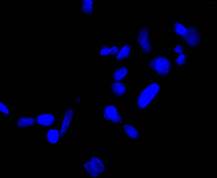

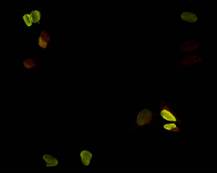

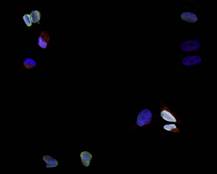

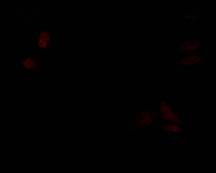

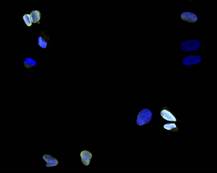

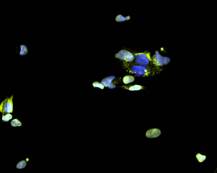

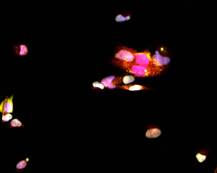

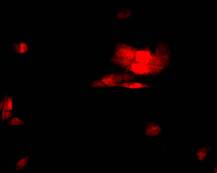

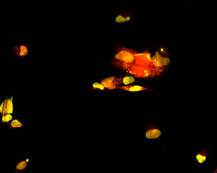

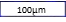

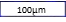

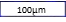

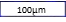

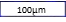

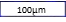

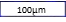

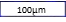

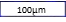

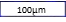

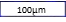

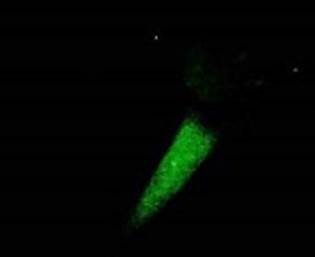

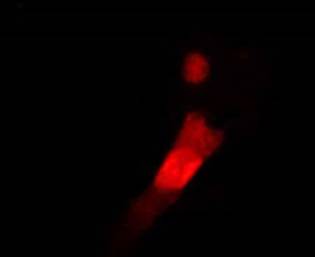

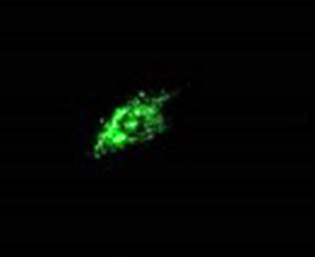

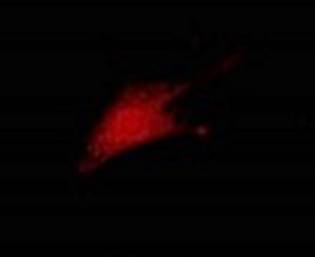

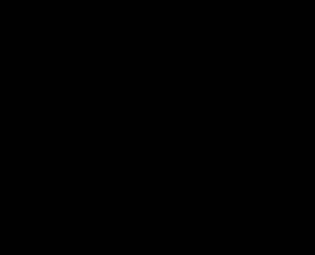

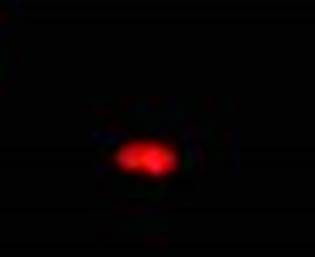
**

100µm

100µm

Metastatic melanoma cell line, hypoxic condition, control IgGs

Metastatic melanoma cell line, normoxic conditions

Metastatic melanoma cell line, hypoxic conditions

**DAPI**

**HMGB1**

**Hif-1α**

**HMGB1**

**Hif-1α**

**DAPI**

**HMGB1**

**Hif-1α**

**HMGB1**

**HIF1α**

**HMGB1**

**HIF1α**

**DAPI**

Metastatic melanoma cell line, hypoxic conditions

Metastatic melanoma cell line, normoxic conditions

b

a

**
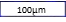

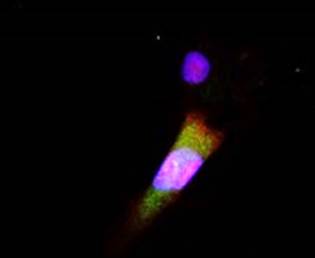

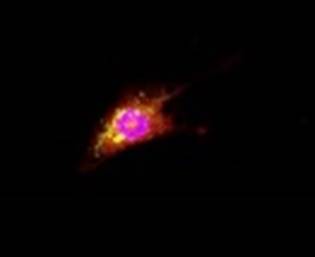

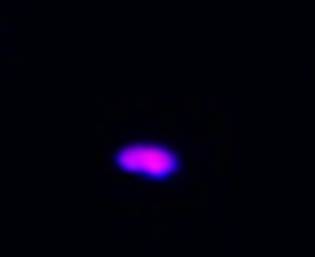

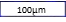

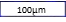

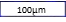

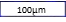

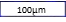

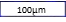

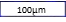

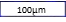

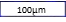
**

**Fig. S1. Hypoxia induces detectable HIF1α and HMGB1 relocalisation in human metastatic melanoma cell lines.** (a) Immunofluorescence co-labelling with anti-HIF1α and anti-HMGB1 antibodies of a metastatic melanoma cell line after 72 hrs in hypoxic conditions or left in normoxia. (b) Higher magnification of melanoma cells cultured in the same conditions as in (a). HIF1a is detectable only when cells are kept under low oxygen and is stabilized in both nucleus and cytosol whereas HMGB1 exhibit different localization from nuclear (normoxia) to cytosolic (hypoxia). (a) and (b) show 2 independent metastatic melanoma cell lines and are representative of experiments repeated 3 times with each.

a

WT-B16

**
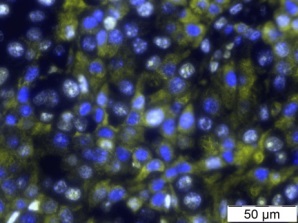

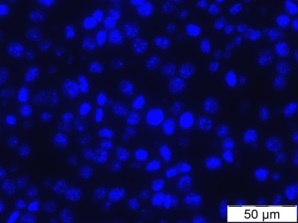

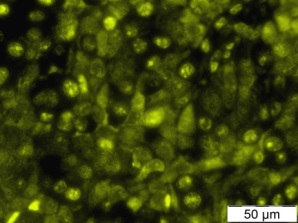

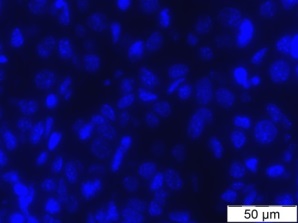

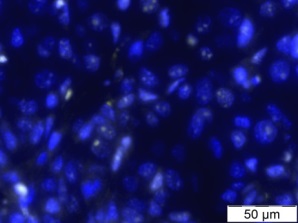

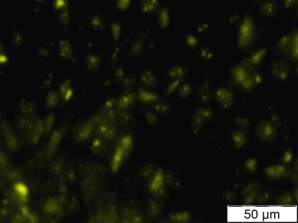

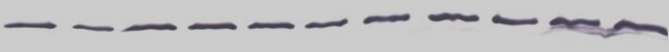

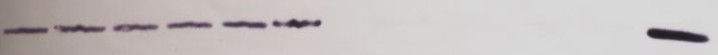
**

D0

D7

D14

D21

D28

D0

D7

D14

D21

D28

c

**DAPI**

**HMGB1**

shHMGB1-B16

**DAPI**

**HMGB1**

shLamin-B16

shLamin

cl1s2

shHMGB1

cl17s5

HMGB1

β-actin

b

1

2

3

4

5

6

1

3

5

7

8

9

11

14

15

16

17

19

1

2

4

5

2

1

Sequence:

Clone:

WT-B16

WT-B16

**Fig. S2. Validation and selection of clones based on HMGB1 knock-down efficiency and stability.** (a) B16 cells were transduced with 2 sequences of shRNA specific to lamin and 4 HMGB1-specific shRNA sequences. Quantitative PCR was performed on *in vitro* expanded transduced/selected cells (puromycin). B16 cells transduced with shRNA specific to lamin (sequence 2) or HMGB1 (sequence 5) were subsequently cloned by the limiting dilution method. Quantitative PCR was performed on *in vitro* expanded transduced/selected clones (puromycin). Results are expressed as 2^-ΔΔCT^ and standardized to wild-type B16 for which a 2^-ΔΔCT^ value of 1 was attributed (dashed line). (b) Cultures of B16 clones transduced with lamin-specific shRNA (clone 1 of sequence 2) or HMGB1 (clone 17 of sequence 5) were harvested, lysed and subjected to western-blot analysis using anti-HMGB1 and anti-β-actin antibodies at the indicated time points. (c) B16 cells transduced with lamin-specific shRNA (clone 1 of sequence 2) or HMGB1 (clone 17 of sequence 5) were injected s.c. to C57BL/6 mice and the resulting tumours were dissected at day 13 and stained with an anti-HMGB1 antibody. Nuclei were visualized using DAPI. Pictures are representative of 7 tumours per group.

**Fig. S3. Exclusion of off-target effects upon transduction of B16 cells with shRNA to lamin.** Wild type and lamin shRNA-expressing B16 cells (cl1s2, control) were injected s.c. into C57BL/6 mice and displayed comparable tumour growth *in vivo* (n=5 mice/group). Representative results of 3 independent experiments are presented.

b

a


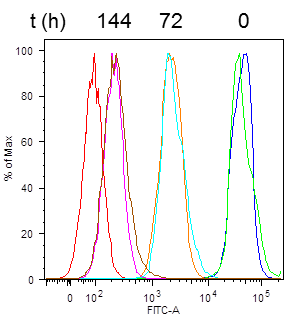


c

d

e

**Fig. S4. The *in vitro* growth properties of B16 cells transduced with shRNA to lamin and HMGB1 are identical.** (a) B16 cells transduced with shRNA specific to lamin or HMGB1 were labelled with CFSE and collected after 72 and 144 h and analysed by flow cytometry. (b) B16 cells transduced with lamin- or HMGB1-specific shRNA were counted over a 15-day culture period. (c) Proliferation of B16 cells transduced with lamin shRNA (white histograms) or HMGB1 shRNA (black histograms) was assessed over a 3-day culture period using the MTT assay. (d) At day 0, 3, 6 and 9, B16 cells transduced with shRNA specific to lamin (shLamin) or HMGB1 (shHMGB1) were harvested, stained with Annexin V and propidium iodide (PI) and analyzed by flow cytometry. Annexin V^+^/PI^+^ cells were considered as late apoptotic. As a positive control, wild-type B16 cells (WT) were treated with the apoptosis inducer staurosporin. (e) Viability of B16 cells transduced with shRNA specific to lamin (white histograms) or HMGB1 (black histograms) was assessed over a 3-day culture period using an LDH release assay. The mean +/- SEM of 3 independent cultures is shown.
